# Elasticity of Mg_3_Bi_2-x_Sb_x_

**DOI:** 10.3390/ma15207161

**Published:** 2022-10-14

**Authors:** Qing Peng, Shuai Zhao, Xiaoze Yuan, Xiao-Jia Chen

**Affiliations:** 1 School of Science, Harbin Institute of Technology, Shenzhen 518055, China; 2 The State Key Laboratory of Nonlinear Mechanics, Institute of Mechanics, Chinese Academy of Sciences, Beijing 100190, China; 3 School of Engineering Sciences, University of Chinese Academy of Sciences, Beijing 100049, China; 4 Department of Modern Mechanics, University of Science and Technology of China, Hefei 230026, China

**Keywords:** thermoelectric materials, Mg_3_Bi_2-x_Sb_x_, elasticity, first-principle calculation

## Abstract

Mg_3_Bi_2-x_Sb_x_ is a promising thermoelectric material working around room temperatures. Compared to electronic and thermoelectric properties, its mechanical properties are of great importance in practical applications but much less understood. Herein, we have systematically studied the elasticity of Mg_3_Bi_2-x_Sb_x_ by means of first-principles calculations with a large supercell of 40 atoms. We demonstrated that the 10-atom-unitcell is undersized with improper electronic structures. With the elastic constants, we have explored the comprehensive elastic features and the three-dimensional distribution of fundamental characteristics of Young’s modulus and Poisson’s ratio and their variation with respect to the Sb content x. We interpolate the variation in terms of the valence electron concentration. We have further examined the hardness, ductility, anisotropicity, and Debye temperatures. The elasticity exhibits strong anisotropy where the maxima are approximately three times larger than the minima for modules. A nearly linear dependence is also observed on the Sb content except x in the vicinity of 0.5. Our atomistic insights on elasticity might be helpful in the material design of thermoelectrics with desirable mechanical properties. Our work could serve as a map for tuning the mechanical properties of Mg_3_Bi_2-x_Sb_x_ and guide the possible synthesizing of novel thermoelectric material.

## 1. Introduction

Materials with the ability to convert electricity into thermal energy are required in the wide application of thermoelectrics [1]. Compared to the state-of-the-art Bi_2_Te_3_ [2] or Ag_2_Se [3] alloys at around room temperature, Mg_3_Bi_2-x_Sb_x_ is much cheaper, free from the expensive elements, but has better thermoelectric performance [4,5,6] and thereby has the promising commercial prospects [7,8]. A figure of merit of zT≈1.5 at 716 K for Mg_3_Bi_1.5_Sb_0.5_ is reported [9]. It is comparable to but cheaper than BiTe alloys, whose, for instance, peak zT of 1.4 at 100 °C is captured [10]. So far, Mg-Bi-Sb materials have garnered increasing attention as their distinguished thermoelectric properties have been reported both by experiments and theories [11,12,13,14]. The thermal, electrical, and thermoelectric properties are extensively studied [15], such as the band topology [16,17,18,19], phonon dynamics [20,21,22,23,24], and topological thermoelectricity of nodal-line semimetal [25,26,27,28]. The intrinsic defects, such as grain boundaries, make the synthesizing of Mg_3_Bi_2-x_Sb_x_ challenging in the past few years. However, Tamaki et al. have observed the prominent of high zT, which is expected to appear with negligible grain boundaries for the high Bi/Sb ratio at three [9]. Mg_3_Bi_2-x_Sb_x_ has a higher carrier mobility and low thermal conductivity arising from the disordered structure [29]. Aside from the microstructures, the intrinsic properties of these site-disordered materials are mainly responsible for their outstanding thermoelectric behaviors.

As one of the fundamental properties, elasticity is required to completely describe the mechanical behavior of this kind of cubic material [30], detailing the elastic stability, elastic anisotropy, ductile manner, and so on, which can surely serve as a reference for the further experimental and theoretical studies and applications on these thermoelectric compounds. First-principles calculations have been widely used to explore the underling physics from the atom-scale perspective. However, the mechanical properties of Mg_3_Bi_2-x_Sb_x_ are much less studied compared to the electronic and thermoelectric properties. The variation of the mechanical properties with respect to the Sb content is still elusive.

In this work, we have systematically investigated the elasticity of Mg_3_Bi_2-x_Sb_x_ via first-principles calculations within the framework of density functional theory. We have compared the results from two models: one within 10-atom-unitcell and the other 40-atom-unitcell. The mechanical response of the Bi/Sb ratio of 3 is scrutinized. Then we studied the influence of Sb potency on the elastic properties. They have a consistent linear trend except for several potencies around 0.5. The three-dimensional distribution of anisotropic Young’s modulus and Poisson’s ratio are illustrated for the Sb content x ranging from 0 to 2. The general trends of hardness, ductility, anisotropicity, and Debye temperatures are further examined.

## 2. Materials and Methods

**Figure 1:**
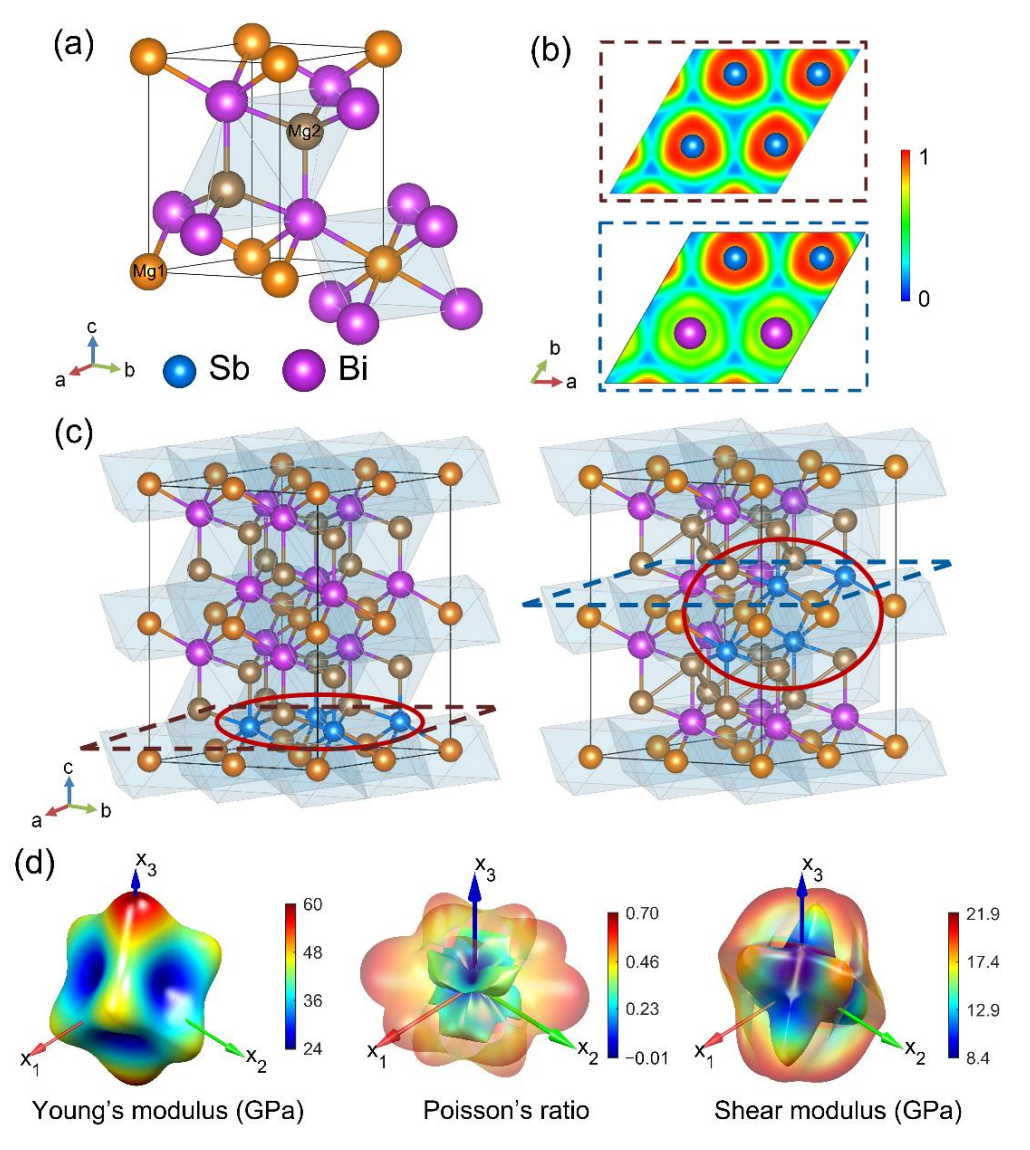
Structures with different sizes of supercell and elastic properties distribution for Mg_3_Bi_1.5_Sb_0.5_. (**a**) Crystalline unit for Mg_3_Bi_2-x_Sb_x_. Bi sites are co-occupied by Bi and Sb atoms coinciding with the proportion. (**b**) Electron location function (ELF) of the same slice for different supercells with 10 atoms (**up**) and 40 atoms (**down**). (**c**) Structures with minimized energy for 10 atoms (**left**) and 40 atoms (**right**). For ease of comparison, the structure of 10 atoms is duplicated by 2 × 2 × 1. The observation slices for ELF in (**b**) are displaced by dashed border. Sb atoms distributions are highlighted by red circles. (**d**) three-dimensional orientation-dependent Young’s modulus, shear modulus, and Poisson’s ratio are derived from the elastic constant combination.

Both Mg_3_Bi_2_ and Mg_3_Sb_2_ have a crystal structure in the trigonal P-3m1 space group with a group number of 164. The primitive unitcell of Mg_3_Bi_2_ contains 3 Mg atoms and 2 Bi atoms. The crystalline structures of Mg_3_Bi_2-x_Sb_x_ (0 ≤ x ≤ 2) adopt the same space group of Mg_3_Bi_2_. The 2 Bi sites are replaced by Bi or Sb atoms according to their stoichiometry, as shown in Figure 1a. The symmetric repetitive within all enumerated 2 × 2 × 2 supercell structures of 40 atoms is eliminated by the open-source program Disorder [31]. These structures are then optimized by rough convergence criteria of first-principles calculations. The structure of minimum energy within all optimized structures at each Sb content x is the representative configuration. For the elastic property calculations, more precise structural optimizations are then used for configurations at each Sb content x. Twelve forced deformations, corresponding to positive and negative micro-strains for six independent components, are applied respectively to the configuration. Elastic constants can be obtained by the corresponding elastic response, manifested as six stresses components from the first-principles results.

Our first-principles calculations are within the framework of Kohn–Sham density functional theory [32] as implemented in the Vienna Ab initio Simulation Package (VASP) [33]. The interactions between atoms are described by Projector Augmented-Wave (PAW) [34] method. Generalized gradient approximation (GGA) developed by Perdew–Burke–Ernzerhof (PBE) [35] was adopted for the exchange-correlation functional. K-meshes are gamma-centered Monkhorst-Pack scheme [36] of 4 × 4 × 2 for structures of 40-atom unitcells and 9 × 9 × 2 for those of 10-atom unitcells. The cutoff energy for plane wave expansion is 260 eV. The order of Methfessel–Paxton method [37] is 1, which is responsible for the partial occupancies for each orbital, and its smearing width is 0.2 eV. The convergence accuracy is set to be 10^−5^ and 10^−8^ eV in the structure searching and configuration optimizing for the electronic self-consistent iterations. Energy convergence criteria of 10^−4^ eV are used for the fast optimization in structure searching, while the force convergence criteria of 10^−3^ eV/Å are set for that of accurate structural optimization. The three-dimensional distribution data is generated by VASPKIT package [38] such as Young’s modulus, Poisson’s ratio, and shear modulus, which is simply expressed in terms of the compliance matrix [39]. The compliance matrix is the inverse matrix of the rotated elastic constant whose coordinates is parallel to the appointed orientations.

## 3. Results

Resembling the bi-components solid solution, the initial-generated structures of the larger supercell are duplicated from the crystalline units, shown in Figure 1a. Mg atoms can be divided into two kinds, denoted as Mg1 (yellow) and Mg2 (brown), according to distinctive polyhedrons of neighbor Bi sites. For Mg1, it is surrounded by six Bi sites, forming an octahedron environment while that of Mg2 is four, possessing a diamond-like tetrahedron environment. Among all enumerated structures of 40 atoms, the symmetric repetitive ones are eliminated. The rest structures are then optimized to search for an energy-minimized structure. The final chosen structure of the lowest energy is used to represent real-world states because the most stable signifies the highest possibility of existence.

For instance, we take the structures of Mg_3_Bi_1.5_Sb_0.5_ as an example since this compound possesses the best thermal-electronic effect. In general, electronic charge density is the most direct measurement of atomic interaction. The difference can be visualized intuitively from the electron location function (ELF) [40,41] of a horizontal plane crossing the most Sb atoms (Figure 1b) and Sb atoms distribution for the configuration of 10-atom unitcell and 40-atom unitcell, respectively (Figure 1c). The maximum of ELF only appears around the Sb ions owing to the smaller radius compared to Bi ions. Thereby, the stronger interactions around the Sb ions are typically deduced from the mechanistic perspective.

Due to the periodic images along the x_1_ and x_2_ axes, which is the intrinsic shortcoming of a small supercell, the Sb atoms take a stratiform distribution for a 10-atom unitcell. The structural stability of different grid arrangements with different ordered ions but the same Sb content, as well as the possible different lattice distortion patterns even with the same ordered ions, cannot be simulated. While in the 2 × 2 × 2 duplicated structure of a 40-atom unitcell, Sb ions accumulate with other Sb ions, giving a trend of precipitation that may trigger differences in the prediction of vast properties, especially the significant thermoelectric properties, to some extent.

We have investigated the elastic properties for Mg_3_Bi_1.5_Sb_0.5_ using a 40-atom unitcell since the structure of the larger supercell is screened from the thermal stability. The 40-atom unitcells are more reasonable than the 10-atom unitcell owing to the larger configurational entropy. The elastic constants are firstly obtained from the DFT calculations. With these elastic constants, the distribution of anisotropic Young’s modulus, shear modulus, and Poisson’s ratio in the three-dimensional Cartesian coordinates are then computed and displayed in Figure 1d. The axes x_1_ and x_3_ are parallel to the lattice vector a and c defined in Figure 1a, respectively.

These elastic properties are extremely orientation-dependent. The maximum Young’s modulus is 60 GPa along the direction of the x_3_ axis, while the minima is 24 GPa within the cubic-shaped faces. For shear modulus and Poisson’s ratio, another investigated direction is needed, which is perpendicular to the normal vector of the plane provided. Thus, a range of values for shear modulus and Poisson’s ratio can be obtained in a specific direction. Merely the maximum and minimum values are what we are interested in, and they are plotted together in Figure 1d (outer transparent surface to be the maxima and inner surface to be the minima) to be easily acquired and clearly compared. For Poisson’s ratio, not only the maximum and minimum values, from −0.01 to 0.70, but also the value ranges for different orientations vary greatly.

For shear modulus, it exhibits six local maximum values, the directions of which are vertical to each other, where the shear resistance is strong in all directions. The maximum value is 21.9 GPa, and the minimum value is 18.0 GPa in these directions. Three of the upper halves make the same angle with x_3_-axis. While on the x_1_–x_2_ plane, it satisfies the trigonal symmetry that midpoints of six edges are in the x_1_–x_2_ plane, corresponding to six apexes of a honeycomb structure. Conversely, the direction, which is in the middle of three adjacent maximum directions that are at 90° to each other, has the weakest stiffness in all directions, with the maxima and minima being 10.7 GPa and 8.4 GPa. For directions not in the vicinity of the above 14 specific directions, they have large shear anisotropy, which represents a large maximum and small minimum shear resistance.

**Figure 2:**
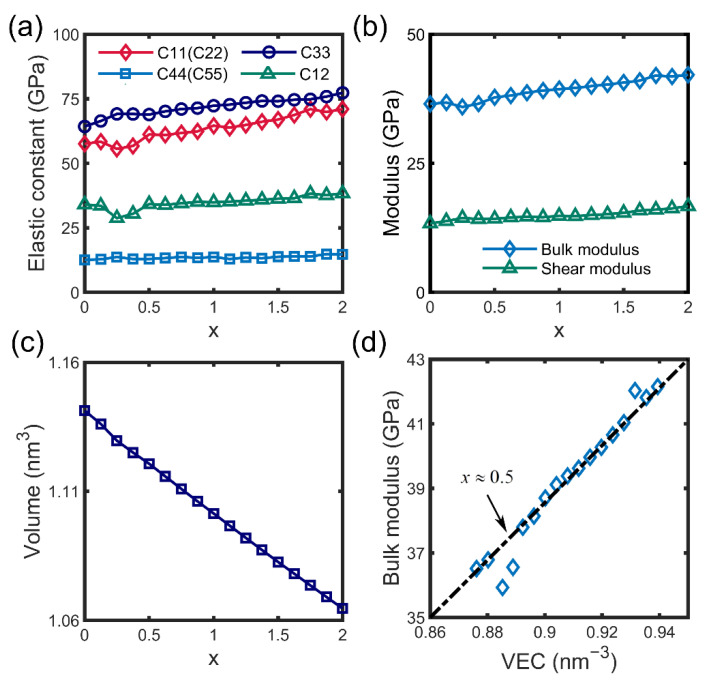
Elastic properties with the increasing of Sb content x. (**a**) The critical elastic constants and (**b**) deduced comprehensive elastic properties of bulk modulus and shear modulus with the change of x from 0 to 2. (**c**) The volume of configuration at each Sb content x which is a presentation of electronic location. (**d**) Linear relation of bulk modulus and the average valence electron concentration (VEC) except for potencies around x ≈ 0.5.

Based on the ability to search for the most stable structures at any Sb content x, mechanical properties strongly depend on the relative concentration of Sb and Bi. We scrutinized the elastic constant combinations at all available Sb contents in our model. The crystalline structure belongs to the trigonal system, possessing eight independent components: C_11_, C_12_, C_13_, C_33_, C_14_, C_15_, C_45_, and C_44_. Owing to the distortion of asymmetry, the lattice is disturbed to a triclinic one considering differences that are not negligible but minor. Thereby, merely the critical elastic constants are plotted in Figure 2a. Across the board, the elastic constants possess a positive correlation function of Sb content x. Overall trends are linear except for the proportion of 0.25 and 0.375, which exhibit abnormal bias.

The comprehensive elastic properties, such as isotropic estimated bulk modulus and shear modulus, can be derived from the elastic constant combination based on the empirical relationship, which is described as:

(1) $$\begin{array}{c} B=\frac{1}{2}\left(B_{V}+B_{R}\right)\\ G=\frac{1}{2}\left(G_{V}+G_{R}\right) \end{array}$$

where Voigt–Reuss–Hill approximation [42,43,44] of the bulk modulus is given as the function of elastic constant *C*_ij_ and its compliance *S*_ij_:

(2a) $$\begin{array}{c} B_{V}=\frac{1}{9}\left(C_{11}+C_{22}+C_{33}\right)+\frac{2}{9}\left(C_{23}+C_{13}+C_{12}\right)\\ B_{R}=\frac{1}{\left[3\left(S_{11}+S_{22}+S_{33}\right)+6\left(S_{23}+S_{13}+S_{12}\right)\right]} \end{array},$$

and that of shear modulus is:

(2b) $$\begin{array}{c} G_{V}=\frac{1}{5}\left[\left(C_{11}+C_{22}+C_{33}\right)-\left(C_{23}+C_{13}+C_{12}\right)\right]+\frac{3}{5}\left(C_{44}+C_{55}+C_{66}\right)\\ G_{R}=\frac{15}{\left\{12\left[\left(S_{11}+S_{22}+S_{33}\right)-\left(S_{23}+S_{13}+S_{12}\right)\right]+9\left(S_{44}+S_{55}+S_{66}\right)\right\}} \end{array}.$$

The curve of bulk modulus and shear modulus border upon a linear relationship depending on the elastic constants in Figure 2b. For the elastic constants of state-of-the-art thermoelectric material of Bi_2_Te_3_, Feng et al. have calculated it by using the first-principles calculation package of Cambridge Serial Total Energy Package (CASTEP) [45]. The bulk modulus and shear modulus, estimated from the elastic constants combinations as well, are 42.0 GPa and 25.9 GPa, respectively. It has double resistance to shearing but a similar bulk modulus compared to Mg_3_Bi_2-x_Sb_x_. The causa essential for these variances of electronic, thermal, and thermoelectric properties is the arrangement of the electron cloud caused by the different occupation of Bi/Sb. Whatever the x is, the total number of valence electrons does not change for the ternary compound Mg_3_Bi_2-x_Sb_x_. Therefore, from the statistical perspective, average valence electron concentration (VEC) has an inverse relationship with the volume, i.e., VEC is of a higher level for the smaller volume.

The volume of supercell at each Sb/Bi ratio is also given in Figure 2c, which possesses an almost consummate linear declining relation of x. The bulk modulus, considered as the representative of the comprehensive elastic property, is thereby intuitively induced to possess a linear degressive function of x. The real tested bulk modulus curve with average VEC, represented by the reciprocal of the structural volume, is plotted in Figure 2d. Same with the trend of elastic constants, anomalous low bulk modulus appears at the VEC in the vicinity of x = 0.5, the special one with prominent thermoelectricity, likely associated with this phenomenon. In this manner, we can consciously enlarge the VEC by doping some other ions to tune up the properties, such as improving the bulk stiffness. This also explained the better thermoelectric performance observed around x = 0.5 after Te and Co doping [29,46], and some similar method can be further applied to the design of functional materials.

**Figure 3:**
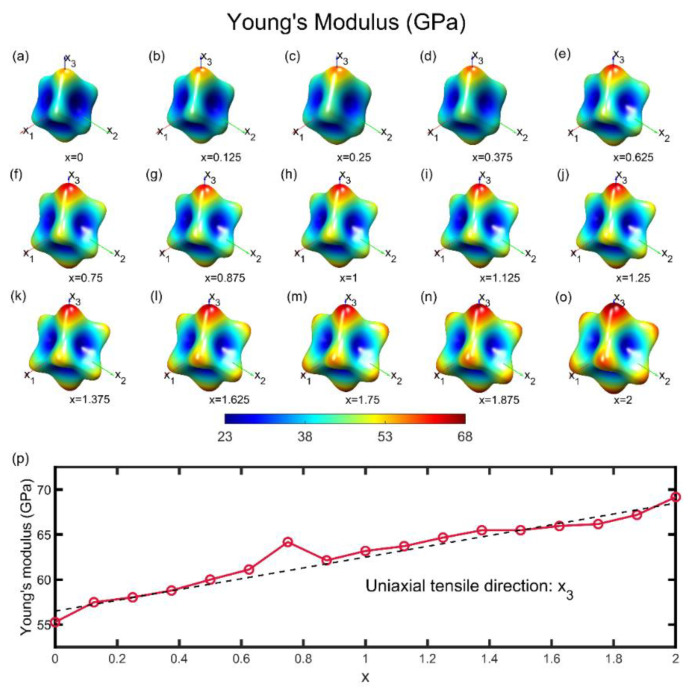
The 3D Young’s modulus distribution at various Sb contents. (**a**–**o**) Direction-dependent Young’s modulus for every potency of Sb at an interval of 0.125, ranging from 0 to 2. (**p**) Young’s modulus along the uniaxial tensile direction of x_3_ as a function of the Sb content x. A linear dependency can be viewed (dashed line).

**Figure 4:**
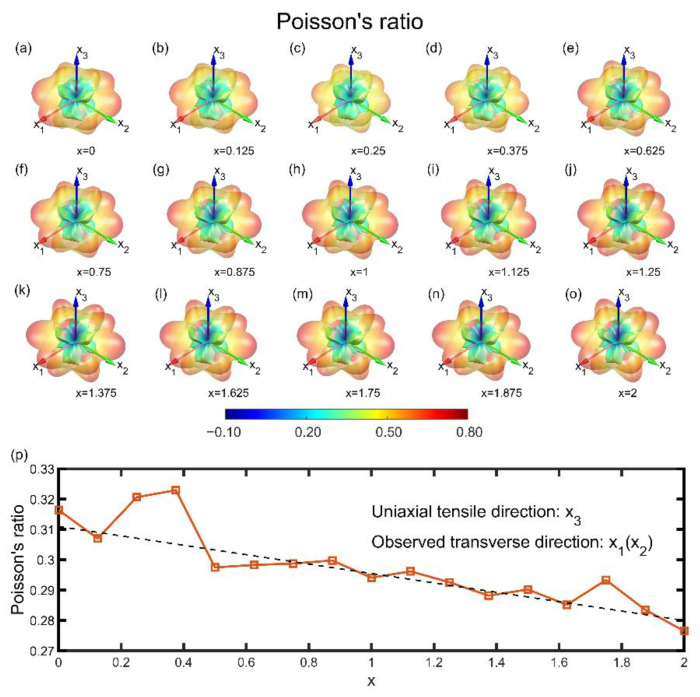
Poisson’s ratio distribution at various Sb contents. (**a**–**o**) Direction-dependent shear modulus at different Sb content x at an interval of 0.125, ranging from 0 to 2. Outer surface denotes the maximum Poisson’s ratio, and inner surface is on the contrary. (**p**) Poisson’s ratio of the tensile direction along x_3_ and observed transverse direction along x_1_ or x_2_ varies with respect to the Sb content x. A linear dependency can be viewed (dashed line).

In the like manner of Mg_3_Bi_1.5_Sb_0.5_, we investigated the stiffness property (Figure 3) and Poisson’s effect (Figure 4) in all directions and all available Sb content x of 40-atom unitcells.

The evolution of direction-dependent Young’s modulus in the three-dimensional space with the growth of Sb’s proportion is shown in Figure 3a–o. For each proportion, Young’s modulus exhibits a distinctive anisotropy where the maximum value is about three times larger than the minimum. Distribution in space is like a cubic shape, whose corner occurs along the x_3_ direction, which denotes the stiffest property. Similarly, the cubic structure is stretched along x_3_ direction. Six planes of the cubic are concave faces because of the low rigidity, whereas, vice versa, eight corners are protuberant. The lattice vector in the x_1_–x_2_ plane is across the midpoints of the edges in this plane where the trigonal symmetry still exists. These distribution characteristics arise from similar lattice structures where the Mg2 ions take the 3 m and −3 m symmetry sites of the original generated structure.

A smooth transition is displayed from purely Bi ions occupied to purely Sb ions occupied with the increasing value of the Sb ions occupying ratio from the Young’s modulus along x_3_ in Figure 3p. In general, Mg_3_Bi_2_ and Mg_3_Sb_2_ exhibit similar Young’s modulus distribution features except for the specific value at eight corners. The maxima value of Young’s modulus grows from 55 GPa to 68 GPa, while the minima value has little change with a fluctuation within the range from 23 to 25 GPa. It is reasonable that Bi and Sb have a similar outer layer valence electron distribution but different atom sizes, which is responsible for the interionic distance. For instance, the lattice parameter of Mg_3_Bi_2_ along the x_1_ direction is 9.45 Å while that of Mg_3_Sb_2_ is 9.20 Å and that along the x_3_ are 14.8 Å and 14.5 Å, respectively. Naturally, larger stiffness is owing to the shorter atomic distance, where higher electronic charge density exists, leading to the stronger interaction between ions.

Poisson’s ratio describes the reverse transverse deformation caused by mechanical loading. For Mg_3_Bi_2-x_Sb_x_ materials, there is no obvious change relationship from the 3D figures with the increase of x, which is different from Young’s modulus and shear modulus. This is because Poisson’s ratio is related to the relative value of the elastic constant, while each component of the elastic constant has an approximately linear increasing trend. It is worth noting that Poisson’s ratio for Mg_3_Bi_2-x_Sb_x_ varies widely for different angles. For the plane vertical to the x_3_ direction, the Poisson’s ratios of all directions on it are small, minima of which are close to 0 or even negative, where the transverse shrinking deformation does not change with the tensile strain, which is rare in conventional alloy materials. In contrast, for all directions within the x_1_–x_2_ plane, the Poisson’s effect spans a lot, although the minimum values are also close to zero.

For six trigonal symmetry directions, two of which are the lattice vectors, exhibit the largest Poisson’s ratio of 0.8. To conclude, as shown in Figure 4p, with the increase of Sb content, the Poisson’s effect does not obtain an obvious improvement except for the proportion of 0.25 and 0.375, as well as other mechanical properties. The innate physical images between the prominent thermoelectricity and well-characterized elastic properties at around 0.5 are still elusive and pending issues.

**Figure 5:**
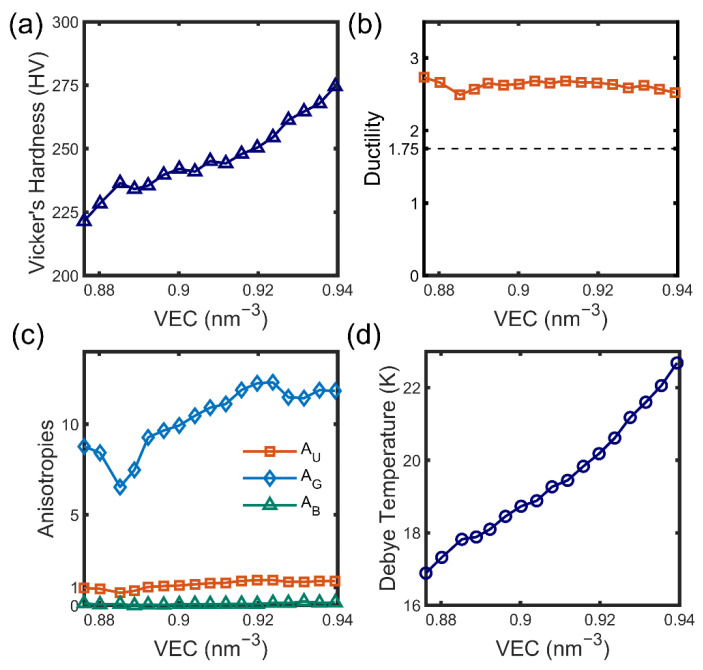
Mechanical and vibrational properties estimated by elastic constants. (**a**) Vicker’s hardness of an empirical model, (**b**) ductility (orange line) predicted from the Pugh ratio (**c**) anisotropies of universal, bulk modulus, and shear modulus, and (**d**) Debye temperature derived from the average sound velocity are presented.

The Vicker’s hardness can be estimated by empirical models from Jiang’s model [47], Teter’s model [48], or Chen’s model [49,50], expressed by:

(3) $$\{\begin{array}{l} H_{E}=0.0608E\\ H_{G}=0.1769G-2.899\\ H_{v}=2{\left(G^{3}/B^{2}\right)}^{0.585}-3 \end{array}$$

and the universal Young’s modulus E is given by E=9GB/(3B+G), whose results are illustrated in Figure 5a. The negative values of the two materials (x = 0 and x = 0.125) predicted from the $H_{v}$ and $H_{G}$ are unphysical and, therefore, not shown here. It implies that the empirical relationship of $H_{v}$ and $H_{G}$ is improper for these compounds. Predictions of Jiang’s models show that the largest VEC has the largest hardness and vice versa. Bi_2_Te_3_ has a much higher hardness of 399 HV as a reference.

Similar to hardness, ductility could be presumably predicted by the ratio between bulk and shear modulus B/G, which is named as Pugh ratio [51]. It is empirically induced that the crystalline is ductile if the Pugh ratio is larger than 1.75 (dashed line in Figure 5b), while the Puff ratio of Bi_2_Te_3_ is 1.62, which is predicted to be brittle in contrast. As a general trend, excluding that of x around 0.5 in Figure 5b, the ductility of Mg_3_Bi_2-x_Sb_x_ exhibits a declining dependence of Sb content x since the Pugh ratio is positively corrected to ductility. Mg_3_Bi_2-x_Sb_x_ behaves in a more brittle manner for x = 0.25 than that of Mg_3_Sb_2_.

The anisotropicity can be described by universal anisotropies $A_{U}$, shear anisotropies $A_{G}$ and bulk anisotropies $A_{B}$ [52,53], whose expression is:

(4) $$\{\begin{array}{l} A_{U}=\frac{5G_{V}}{G_{R}}+\frac{B_{V}}{B_{R}}-6\\ A_{G}=\frac{G_{V}-G_{R}}{G_{V}+G_{R}}\times 100\\ A_{B}=\frac{B_{V}-B_{R}}{B_{V}+B_{R}}\times 100 \end{array}$$

which quantitatively provides insights into the orientation-dependence anisotropic properties. As shown in Figure 5c, the calculated $A_{B}$ is close to 0. Shear anisotropies $A_{G}$ are much larger for Mg_3_Bi_2-x_Sb_x,_ which reveals that the bulk properties are isotropic and shear resistances are on the contrary, which agrees with the aforementioned orientation-dependent shear modulus in Figure 1d; however, Bi_2_Te_3_ does not, whose $A_{B}$ is 1.58, larger than 1 as well, declaring its stronger anisotropy. The universal anisotropies $A_{U}$ suggest the strong directional dependence of Mg_3_Bi_2-x_Sb_x_ compared to other alloys such as Zr_5_Sn_3_ and Zr_5_Sn_3_X (X = B, Nb, and Sn), whose holistic $A_{U}$ are much less than 0.5 [53]. The anisotropy is strengthened with more Sb content whose $A_{U}$ increases slightly with respect to an increase in VEC.

Debye temperature, an important evaluation of thermodynamic properties, is calculated by the average sound velocity, which can be estimated by elastic constants [54]. Proportional to average sound velocity $v_{m}$, the Debye temperature Θ is estimated by:

(5) $$\Theta =\frac{h}{k_{B}}{\left(\frac{3q}{4\pi }\frac{N\rho }{M}\right)}^{\frac{1}{3}}v_{m}$$

where the h, N, and $k_{B}$ are the Planck constant, Avogadro constant, and Boltzmann constant, respectively, ρ is the mass density, q is the number of atoms in the supercell, and M is the molecular weight of all atoms in the supercell. The average sound velocity is proposed by shear sound velocity $v_{s}$ and longitudinal sound velocity $v_{l}$:

(6) $$v_{m}={\left[\frac{1}{3}\left(\frac{2}{v_{s}{}^{3}}+\frac{1}{v_{l}{}^{3}}\right)\right]}^{-\frac{1}{3}}$$

where $v_{s}=\sqrt{G/\rho }$ and $v_{l}=\sqrt{\left(B+\frac{3}{4}G\right)/\rho }$. It is obtained from Figure 5d that Debye temperature grows from the minimum 16.8K of Mg_3_Bi_2_ to the maximum 22.7 K of Mg_3_Sb_2_, similar to that of Bi_2_Te_3_, which is predicted to be 23.2 K. Debye temperature possess a positive function of VEC indicating that the melting point is higher with tighter bonding.

## 4. Conclusions

We have systematically investigated the elasticity of Mg_3_Bi_2-x_Sb_x_ (0 ≤ x ≤ 2) by means of DFT calculations. For the same concentration, thus the same chemical formula and stoichiometry, the atomistic configurations are different at unicells that differ in size. The distinct configurations can be viewed directly from the structures and electron location function distributions for Mg_3_Bi_1.5_Sb_0.5_ with 10 atoms and 40 atoms. The structure has impacts on the properties, and we have examined the effect of the unitcell size on the elasticity of the Mg_3_Bi_2-x_Sb_x_, taking Mg_3_Bi_1.5_Sb_0.5_ as an example. Anisotropic elastic properties are discovered from the three-dimensional visualization of modules, whose maxima values are about three times larger than the minima, and Poisson’s ratios possess a wide variation range with different orientations.

The elasticity variety of the Sb/Bi ratio is also systematically explored for the ternary compound Mg_3_Bi_2-x_Sb_x_. From the single elastic constant component to comprehensive bulk modulus, the elastic properties exhibit a linear dependency of the valence electric concentration VEC, except for that of x around 0.5, which is reported to have the merit zT 1.5 at room temperature. The distribution of Young’s modulus and Poisson’s ratio in the three-dimensional Cartesian coordinates of all available x are analyzed, as well as other estimated material properties, including hardness, ductility, anisotropicity, and Debye temperature. The abnormal distribution of Poisson’s ratio at x = 0.25 and x = 0.375 can be visualized directly. These phenomena may be of great importance in finding the underling physical rules for Mg_3_Bi_1.5_Sb_0.5,_ which might give important references for the further discovery of novel functional materials with higher zT.

## Data Availability

Not applicable.

## References

[1] Bell L.E. (2008). Cooling, heating, generating power, and recovering waste heat with thermoelectric systems. Science.

[2] Ao D.W., Liu W.D., Chen Y.X., Wei M., Jabar B., Li F., Shi X.L., Zheng Z.H., Liang G.X., Zhang X.H. (2022). Novel Thermal Diffusion Temperature Engineering Leading to High Thermoelectric Performance in Bi_2_Te_3_-Based Flexible Thin-Films. Adv. Sci..

[3] Yang Q., Yang S., Qiu P., Peng L., Wei T.-R., Zhang Z., Shi X., Chen L. (2022). Flexible thermoelectrics based on ductile semiconductors. Science.

[4] Imasato K., Kang S.D., Ohno S., Snyder G.J. (2018). Band engineering in Mg_3_Sb_2_ by alloying with Mg_3_Bi_2_ for enhanced thermoelectric performance. Mater. Horiz..

[5] Zhang F., Chen C., Yao H., Bai F., Yin L., Li X., Li S., Xue W., Wang Y., Cao F. (2020). High-performance N-type Mg_3_Sb_2_ towards thermoelectric application near room temperature. Adv. Funct. Mater..

[6] Shi X., Sun C., Bu Z., Zhang X., Wu Y., Lin S., Li W., Faghaninia A., Jain A., Pei Y. (2019). Revelation of inherently high mobility enables Mg_3_Sb_2_ as a sustainable alternative to n-Bi_2_Te_3_ thermoelectrics. Adv. Sci..

[7] Zhang J., Song L., Pedersen S.H., Yin H., Hung L.T., Iversen B.B. (2017). Discovery of high-performance low-cost n-type Mg_3_Sb_2_-based thermoelectric materials with multi-valley conduction bands. Nat. Commun..

[8] Pan Y., Yao M., Hong X., Zhu Y., Fan F., Imasato K., He Y., Hess C., Fink J., Yang J. (2020). Mg_3_(Bi,Sb)_2_ single crystals towards high thermoelectric performance. Energy Environ. Sci..

[9] Tamaki H., Sato H.K., Kanno T. (2016). Isotropic conduction network and defect chemistry in Mg_3+δ_Sb_2−_ based layered zintl compounds with high thermoelectric performance. Adv. Mater..

[10] Poudel B., Hao Q., Ma Y., Lan Y., Minnich A., Yu B., Yan X., Wang D., Muto A., Vashaee D. (2008). High-thermoelectric performance of nanostructured bismuth antimony telluride bulk alloys. Science.

[11] Imasato K., Kang S.D., Snyder G.J. (2019). Exceptional thermoelectric performance in Mg_3_Sb_0.6_Bi_1.4_ for low-grade waste heat recovery. Energy Environ. Sci..

[12] Imasato K., Wood M., Kuo J.J., Snyder G.J. (2018). Improved stability and high thermoelectric performance through cation site doping in n-type La-doped Mg_3_Sb_1.5_Bi_0.5_. J. Mater. Chem. A.

[13] Wood M., Kuo J.J., Imasato K., Snyder G.J. (2019). Improvement of Low-Temperature zT in a Mg_3_Sb_2_–Mg_3_Bi_2_ Solid Solution via Mg-Vapor Annealing. Adv. Mater..

[14] Zhang J., Song L., Iversen B.B. (2019). Insights into the design of thermoelectric Mg_3_Sb_2_ and its analogs by combining theory and experiment. NPJ Comput. Mater..

[15] Ullah M., Murtaza G., Ramay S.M., Mahmood A. (2017). Structural, electronic, optical and thermoelectric properties of Mg_3_X_2_ (X = N, P, As, Sb, Bi) compounds. Mater. Res. Bull..

[16] Yu R., Fang Z., Dai X., Weng H. (2017). Topological nodal line semimetals predicted from first-principles calculations. Front. Phys..

[17] Teshome T., Datta A. (2019). Topological phase transition in Sb_2_Mg_3_ assisted by strain. ACS Omega.

[18] Li S., Yu Z.-M., Liu Y., Guan S., Wang S.-S., Zhang X., Yao Y., Yang S.A. (2017). Type-II nodal loops: Theory and material realization. Phys. Rev. B.

[19] Zilberberg O., Lu H.-Z., Chen W. (2019). Weak Localization and Antilocalization in Nodal-Line Semimetals: Dimensionality and Topological Effects. Phys. Rev. Lett..

[20] Zhu Y., Liu J., Wei B., Xu S., Song Y., Wang X., Xia T.-L., Chen J., Snyder G., Hong J. (2022). Giant phonon anharmonicity driven by the asymmetric lone pairs in Mg_3_Bi_2_. Mater. Today Phys..

[21] Xin J., Li G., Auffermann G., Borrmann H., Schnelle W., Gooth J., Zhao X., Zhu T., Felser C., Fu C. (2018). Growth and transport properties of Mg_3_X_2_ (X = Sb, Bi) single crystals. Mater. Today Phys..

[22] Agne M.T., Imasato K., Anand S., Lee K., Bux S.K., Zevalkink A., Rettie A.J., Chung D.Y., Kanatzidis M.G., Snyder G.J. (2018). Heat capacity of Mg_3_Sb_2_, Mg_3_Bi_2_, and their alloys at high temperature. Mater. Today Phys..

[23] Ding J., Lanigan-Atkins T., Calderón-Cueva M., Banerjee A., Abernathy D.L., Said A., Zevalkink A., Delaire O. (2021). Soft anharmonic phonons and ultralow thermal conductivity in Mg_3_(Sb,Bi)_2_ thermoelectrics. Sci. Adv..

[24] Peng W., Petretto G., Rignanese G.-M., Hautier G., Zevalkink A. (2018). An unlikely route to low lattice thermal conductivity: Small atoms in a simple layered structure. Joule.

[25] Gooth J., Schierning G., Felser C., Nielsch K. (2018). Quantum materials for thermoelectricity. MRS Bull..

[26] Li M., Chen G. (2020). Thermal transport for probing quantum materials. MRS Bull..

[27] Barati S., Abedinpour S.H. (2020). Thermoelectric response of nodal-line semimetals: Probing the Fermi surface topology. Phys. Rev. B.

[28] Hosoi M., Tateishi I., Matsuura H., Ogata M. (2022). Thin films of topological nodal line semimetals as a candidate for efficient thermoelectric converters. Phys. Rev. B.

[29] Kanno T., Tamaki H., Yoshiya M., Uchiyama H., Maki S., Takata M., Miyazaki Y. (2021). High-Density Frenkel Defects as Origin of N-Type Thermoelectric Performance and Low Thermal Conductivity in Mg_3_Sb_2_-Based Materials. Adv. Funct. Mater..

[30] Arif M., Murtaza G., Ali R., Khenata R., Takagiwa Y., Muzammil M., Omran S.B. (2016). Elastic and electro-optical properties of XYZ (X = Li, Na and K; Y = Mg; Z = N, P, As, Sb and Bi) compounds. Indian J. Phys..

[31] Lian J.-C., Wu H.-Y., Huang W.-Q., Hu W., Huang G.-F. (2020). Algorithm for generating irreducible site-occupancy configurations. Phys. Rev. B.

[32] Kohn W., Sham L.J. (1965). Self-consistent equations including exchange and correlation effects. Phys. Rev..

[33] Kresse G., Furthmüller J. (1996). Efficiency of ab-initio total energy calculations for metals and semiconductors using a plane-wave basis set. Comput. Mater. Sci..

[34] Blöchl P.E. (1994). Projector augmented-wave method. Phys. Rev. B.

[35] Perdew J.P., Burke K., Ernzerhof M. (1996). Generalized gradient approximation made simple. Phys. Rev. Lett..

[36] Monkhorst H.J., Pack J.D. (1976). Special points for Brillouin-zone integrations. Phys. Rev. B.

[37] Methfessel M., Paxton A. (1989). High-precision sampling for Brillouin-zone integration in metals. Phys. Rev. B.

[38] Wang V., Xu N., Liu J.-C., Tang G., Geng W.-T. (2021). VASPKIT: A user-friendly interface facilitating high-throughput computing and analysis using VASP code. Comput. Phys. Commun..

[39] Marmier A., Lethbridge Z.A., Walton R.I., Smith C.W., Parker S.C., Evans K.E. (2010). ElAM: A computer program for the analysis and representation of anisotropic elastic properties. Comput. Phys. Commun..

[40] Becke A.D., Edgecombe K.E. (1990). A simple measure of electron localization in atomic and molecular systems. J. Chem. Phys..

[41] Peng Q., De S. (2012). Tunable band gaps of mono-layer hexagonal BNC heterostructures. Phys. E.

[42] Reuß A. (1929). Berechnung der fließgrenze von mischkristallen auf grund der plastizitätsbedingung für einkristalle. ZAMM-J. Appl. Math. Mech./Zeitschrift für Angewandte Mathematik und Mechanik.

[43] Hill R. (1952). The elastic behaviour of a crystalline aggregate. Proc. Phys. Soc. Sect. A.

[44] Voigt W. (1908). Lehrbuch der Kristallphysik.

[45] Feng S., Li S., Fu H. (2014). First-principle calculation and quasi-harmonic Debye model prediction for elastic and thermodynamic properties of Bi_2_Te_3_. Comput. Mater. Sci..

[46] Chen Y., Wang C., Ma Z., Li L., Li S., Wang J. (2021). Improved thermoelectric performance of n-type Mg_3_Sb_2_–Mg_3_Bi_2_ alloy with Co element doping. Curr. Appl. Phys..

[47] Jiang X., Zhao J., Wu A., Bai Y., Jiang X. (2010). Mechanical and electronic properties of B_12_-based ternary crystals of orthorhombic phase. J. Phys. Condens. Matter.

[48] Teter D.M. (1998). Computational alchemy: The search for new superhard materials. MRS Bull..

[49] Chen X.-Q., Niu H., Li D., Li Y. (2011). Modeling hardness of polycrystalline materials and bulk metallic glasses. Intermetallics.

[50] Ouyang Y., Chen H., Tao X., Gao F., Peng Q., Du Y. (2018). A first-principles study of the structural, mechanical and electronic properties of precipitates of Al_2_Cu in Al–Cu alloys. Phys. Chem. Chem. Phys..

[51] Pugh S. (1954). Relations between the elastic moduli and the plastic properties of polycrystalline pure metals. Lond. Edinb. Dublin Philos. Mag. J. Sci..

[52] Ravindran P., Fast L., Korzhavyi P.A., Johansson B., Wills J., Eriksson O. (1998). Density functional theory for calculation of elastic properties of orthorhombic crystals: Application to TiSi_2_. J. Appl. Phys..

[53] Chen H., Cao Y., Liu K., Tao X., Zhou Y., Ouyang Y., Gao F., Du Y., Peng Q. (2019). Stability and physical properties tuning via interstitials chemical engineering of Zr_5_Sn_3_: A first-principles study. J. Mater. Sci..

[54] Anderson O.L. (1963). A simplified method for calculating the Debye temperature from elastic constants. J. Phys. Chem. Solids.

